# Guided extraction of genome-scale metabolic models for the integration and analysis of omics data

**DOI:** 10.1016/j.csbj.2021.06.009

**Published:** 2021-06-08

**Authors:** Andrew Walakira, Damjana Rozman, Tadeja Režen, Miha Mraz, Miha Moškon

**Affiliations:** a Centre for Functional Genomics and Bio-Chips, Institute for Biochemistry and Molecular Genetics, Faculty of Medicine, University of Ljubljana, Ljubljana, Slovenia; b Faculty of Computer and Information Science, University of Ljubljana, Ljubljana, Slovenia

**Keywords:** Genome-scale metabolic model, Model extraction methods, Context-specific metabolic model, Omics data integration, Subsystem enrichment analysis, Model interpretability

## Abstract

Omics data can be integrated into a reference model using various model extraction methods (MEMs) to yield context-specific genome-scale metabolic models (GEMs). How to chose the appropriate MEM, thresholding rule and threshold remains a challenge. We integrated mouse transcriptomic data from a *Cyp51* knockout mice diet experiment (GSE58271) using five MEMs (GIMME, iMAT, FASTCORE, INIT an tINIT) in a combination with a recently published mouse GEM iMM1865. Except for INIT and tINIT, the size of extracted models varied with the MEM used (t-test: p-value <0.001). The Jaccard index of iMAT models ranged from 0.27 to 1.0. Out of the three factors under study in the experiment (diet, gender and genotype), gender explained most of the variability (>90%) in PC1 for FASTCORE. In iMAT, each of the three factors explained less than 40% of the variability within PC1, PC2 and PC3. Among all the MEMs, FASTCORE captured the most of the true variability in the data by clustering samples by gender. Our results show that for the efficient use of MEMs in the context of omics data integration and analysis, one should apply various MEMs, thresholding rules, and thresholding values to select the MEM and its configuration that best captures the true variability in the data. This selection can be guided by the methodology as proposed and used in this paper. Moreover, we describe certain approaches that can be used to analyse the results obtained with the selected MEM and to put these results in a biological context.

## Highlights

• Several challenges must be addressed when performing context-specific model extraction.
• We propose a framework for efficient extraction/analysis of genome scale metabolic models.
• We highlight the impact of choice of model extraction method (MEM) on the output models.
• We highlight the impact of thresholding rules and values on the output models.
• Cyp51 knockout mice diet experiment data are used as a case study.

## 1 Introduction

The advent of high-throughput technologies generate large volumes of omics data hence making it possible to study organisms at the cellular level. These studies have enabled a better understanding of the underlying biological processes of many diseases such as diabetes [1], [2] and asthma [3], yielded useful products such as insulin [4], [5] and antibiotics [6], [7], and also improved production of commercial products such as wines [8]. However, there is still a big knowledge gap in the finer details of how organisms, unicellular or multicellular, are able to maintain life and how disruptions at the molecular level affect their phenotypes.

The phenotypic characteristics of an organism are determined by intricately connected reactions and consequently pathways that generate energy and other forms of biological products necessary to sustain life and organise organismal development. These pathways are connected to form and function as biological systems and thus it is vital to study organisms at system level. The degree of complexity of biological systems differs greatly among organisms, for example, humans are far more complex than *Drosophila melanogaster* despite the latter’s importance in modelling human diseases [9], [10], [11]. It is extremely difficult, or even impossible for higher level organisms, to study the entirety of their pathways either *in vitro* or *in vivo*. However, mathematical tools such as genome-scale models can be used to gain insight into how these biological systems function [12].

Genome-scale metabolic models (GEMs) are increasingly becoming a popular tool for studying biological processes *in silico*[13], [14], [15]. GEMs are formulated to contain all known biochemical reactions involved in maintaining the living state of a cell or an organism (metabolism). Context-specific metabolic models (also known as tissue-specific models) are GEMs in which inactive reactions for a given condition (context) are removed [16] and thus represent the context better since not all possible reactions are active in different cell types and/or in different contexts. Moreover, GEMs can be used to perform *in silico* studies and observe the dynamical response of the system in a given condition using computer simulations. As such, they provide better understanding of the organism as a functional system. One of the main problems of using GEMs in a combination with omics data lies in the complexity of the obtained models and the number of models produced. The obtained results are thus difficult to interpret in the context of biology. Moreover, it is hard to select an appropriate model extraction method (MEM) for a specific dataset. When the appropriate MEM is selected, it also needs to be configured, e.g., gene activity thresholds and thresholding rules need to be defined [17], [18] to yield accurate and biologically relevant results.

We aim to address these problems by suggesting a methodology for performing analyses of omics data using GEMs in combination with different MEMs. This methodology will serve as an essential step towards the development of a pipeline that will automatically select a suitable MEM for a specific dataset, perform its configuration, and extract the models. Moreover, such a pipeline could provide its users with a set of publication-ready figures and tables, describing the results of simulations performed upon the derived models as well as the results of different statistical and enrichment analyses ready for a straightforward interpretation.

## 2 Materials and methods

In this section, we describe how a GEM is reconstructed from available biological information to produce a reference model. This is followed by a summary of the data integration algorithms that were applied in our analysis. Finally, we describe our analysis of the dataset used in our case study, namely, mouse diet experiment data.

### 2.1 Genome-scale metabolic models: from reconstruction to simulation

The construction of GEMs can be summarised in four main steps as described by Feist and colleagues [19]. First, a draft reconstruction of the biological network of an organism is extracted using information about reactions, enzymes, and pathways from databases such as KEGG, BRENDA, etc. The second step is the manual curation of the reconstructed draft model. This involves checking and filling the gaps and correcting misplaced reactions. Here, organism-specific databases and literature are used. Computational algorithms such as GAUGE [20], FastGapFill [21], and FBA-Gap [22] can also be applied. The third step is the conversion of the reconstructed model into a mathematical representation that can be used for subsequent simulations. The final step is the refining, validation, and application of the model to inform decisions. Here, the model outputs are compared with known information to confirm consistency. Model validation can be done by testing how well a model performs a set of tasks, comparing the simulation results with experimental data for a particular objective such as growth and use of gene essentiality analysis i.e. identification of genes that are required for survival of an organism [23].

The most efficient approach for prediction of phenotype from genotype using GEMs is constraint-based modelling [24], [25]. Here, it is common to assume that the system is in a steady state, i.e., the concentrations of all metabolites involved are constant (see Eq. 1). Constraint-based modelling is focused on predicting flux distributions while optimising a selected cellular function (or a set of functions). Flux Balance Analysis (FBA) is the most widely used technique to predict flux distributions in GEMs [26], [27]. It requires a mathematical representation of the model in the form of a stoichiometric matrix *S* with rows representing metabolites and columns representing reactions. We can evaluate a vector *v* of fluxes through the observed metabolic reactions constrained by the upper and lower flux bounds by using the equation

(1) $$\begin{array}{cc} \overset{N}{\underset{j=1}{\sum }} & S_{\mathrm{ij}}\cdot v_{j}=0,\\ \forall i\in & \left\{1,2,\ldots ,M\right\},\\ \forall j\in & \left\{1,2,\ldots ,N\right\}, \end{array}$$

where *M* is the number of observed metabolites and *N* the number of observed reactions. FBA reduces the problem to a linear program, hence lowering the computational requirements involved [26]. However, there are certain limitations. First, FBA does not yield a unique solution and is highly dependent on the choice of the objective function, i.e., a description of the phenotype relevant to the problem being studied [28]. While in some cases biomass optimisation is a plausible biological objective (e.g., cancer cells, cell lines, single cell organisms), different optimisation criteria need to be applied depending on the question of interest. How to select an appropriate objective function is an unanswered question. Algorithms that can select the objective function automatically have been proposed [29]. There is also increasing evidence that multiple objective functions are required to allow metabolic flexibility and improve accuracy of the model [30], [31]. The other challenges are that FBA is dependent on the choice of the solver (used to solve the set of linear equations) and the quality of reconstruction [27], [32]. Variations of FBA have been proposed to ease these limitations for example, parsimonious FBA (pFBA) [33] and Flux Variability Analysis (FVA) [34], among others [27], [35]. Another method for predicting metabolic fluxes is flux sampling, which can be used to estimate probability distributions of reaction fluxes without assuming any particular cellular objective [36], [37].

### 2.2 Computational approaches for experimental data integration

Model extraction methods can be classified as members of a “GIMME-like” family which minimises flux through reactions associated with low gene expression, an “iMAT-like” family which finds an optimal trade-off between keeping reactions whose genes are highly expressed and removing reactions associated with low gene expression, and an “MBA-like” family which retains a selected set of core reactions [16], [38]. Below is a summary of the data integration algorithms that were used in this analysis.

#### 2.2.1 GIMME

Gene Inactivity Moderated by Metabolism and Expression (GIMME) [16], [39], [40] uses gene expression data and one or more objective functions to produce a context-specific model. The GIMME algorithm takes three inputs, i.e., expression data, genome scale reconstruction, and one or more required metabolic functionalities (RMFs) that the cell is expected to achieve. The algorithm first finds a flux distribution that optimises the given objective(s) and then minimises the use of inactive reactions (reactions whose expression is below a predefined threshold). The expression data is used directly as weights in the objective function. A threshold is used to determine if a weight in the objective is positive or negative. A weight of zero is assigned to reactions without expression data. The method yields an inconsistency score i.e (flux*(threshold-data)), a score that shows the disagreement between the expression data and the metabolic objective function. The normalised version of this score shows how well each gene in the expression data agrees with a particular metabolic function. GIMME has been successfully used in studies such as [41] aimed at understanding the impact of drought stress on *Arabidopsis thaliana*.

#### 2.2.2 iMAT

The Integrative Metabolic Analysis Tool (iMAT) [42] takes three-valued expression data as inputs. The data are categorised as lowly, moderately, or highly expressed and coded as −1, 0, and 1, respectively. A Boolean gene-to-reaction mapping is used to identify the state of a reaction, i.e., if the genes encoding enzymes of the reaction are low, moderate or highly expressed. This leads to classifying the reactions in the model as either highly or lowly expressed. This is followed by finding the steady state flux distribution that satisfies the stoichiometric and dynamic constraints and maximises the number of reactions whose activity is consistent with their expression state. A reaction is considered active if it carries a significant positive flux (or negative flux for reversible reactions) that is greater than a threshold. A reaction is inactive if it carries a flux of zero (0). The algorithm returns a vector showing the predicted activity state (fluxes) of each reaction. iMAT performs a pathway enrichment analysis and identifies up- and down-regulated genes thus shedding light on the active pathways in the conditions under study.

#### 2.2.3 FASTCORE

FASTCORE [43] is a data integration algorithm that accepts a core set of reactions that are known to be active in regard to the context under study. The core set of reactions can be determined by considering reactions in which highly expressed genes (genes whose expression level is above a predefined threshold) are involved. This is followed by a search for a flux consistent subnetwork, i.e., a network in which each reaction has a non zero flux in at least one feasible flux distribution. Such subnetwork presents a context-specific model which contains no blocked reactions.

#### 2.2.4 INIT and tINIT

Intergrative Network Inference for Tissues (INIT) [44] formulates a mixed integer-linear problem (MILP) designed to use data from the Human Protein Atlas (HPA) and other omics data as inputs. INIT does not apply a strict steady state assumption for all internal metabolites. Instead it allows a small positive net production of metabolites which are given positive weights in the optimization. Consequently, all reactions in the resulting model are able to carry flux. The algorithm produces networks that are snapshots of active metabolism [45]. tINIT is the task-driven version of INIT. Here, a set of tasks that must be carried out by the resulting model are defined first and then followed by the INIT algorithm.

### 2.3 Case study using gene expression data from *Cyp51* knockout mice

The mouse gene expression dataset was downloaded from the GEO database (accession number GSE58271) and processed to obtain normalised gene expression values. Briefly, this dataset was generated from a study in which the mice were divided into three groups and fed on three diets i.e. low fat without cholesterol (LFnC), high fat without cholesterol (HFnC) and high fat with cholesterol (HFC). Each diet group contained both wild type and the *Cyp51* knockout genotype in female and male mice. The detailed description of the dataset can be found in [46].

**Fig. 1:**
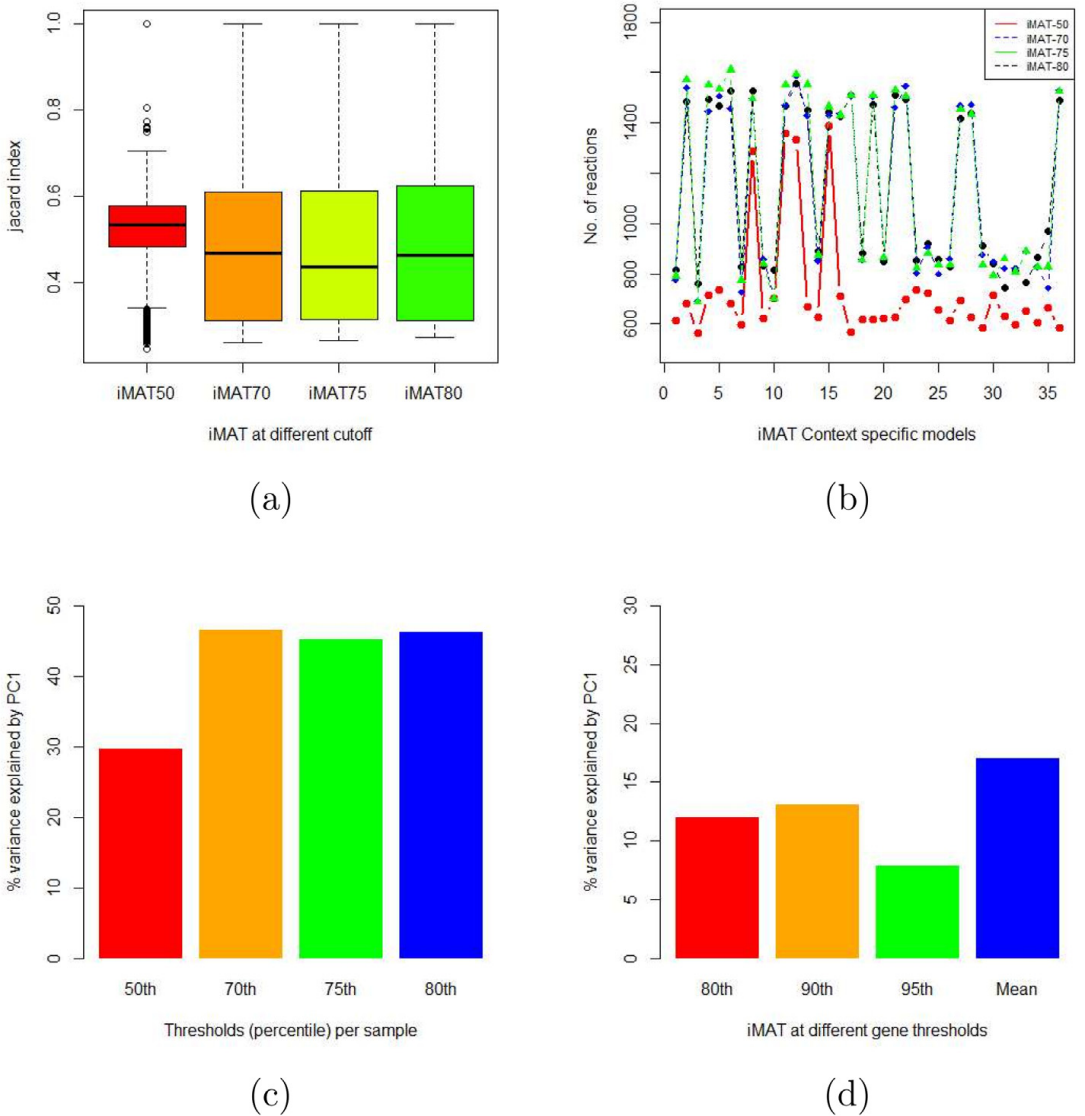
Analysis of iMAT derived models extracted using different thresholding rules and values. Fig. 1 (a), (b), and (c) present the analysis of the models extracted by thresholding per sample, where figure (a) shows the Jaccard index from the pairwise comparisons of all models for each threshold, figure (b) how these models are varied in size, and figure (c) the percentage of variance explained by PC1 for each threshold. Figure (d) shows the variance explained by PC1 in the models extracted when thresholding is performed per gene. PC1: first prin.cipal component.

Extraction of context-specific models was performed in Matlab R2019b (MathWorks Inc., Natick, Massachusetts, USA) using normalised gene expression data and each of the model extraction methods (MEMs) above as described by their respective authors. A recently published mouse model, iMM1865 was used as a reference model. This model has 10612 reactions, 5839 metabolites and 93 subsystems and has no dead-end metabolites or blocked reactions [47]. Highly expressed genes for model extraction were determined by setting the threshold values at the $50^{\mathrm{th}},70^{\mathrm{th}},75^{\mathrm{th}}$, and $80^{\mathrm{th}}$ percentiles of the normalised gene expression data per sample. The rationale of setting this threshold per sample is that all individuals are biologically distinct even when under similar conditions. They yield unique expression patterns implying that their set of highly expressed genes may differ because of these intricate biological differences. Additionally, we assessed the impact of using thresholds set per each gene separately, i.e., thresholds defined within a gene. Here, we considered the $80^{\mathrm{th}},90^{\mathrm{th}},95^{\mathrm{th}}$ percentile and the mean for each gene within the observed population. The thresholds were chosen based on the trend of the variance explained by the first principal component (PC1) (Fig. 1c and d). The extracted models from each algorithm were compared with one another (pairwise comparison) using the Jaccard index (distance) metric [48] to identify the distance between models extracted under the same conditions.

Principal component analysis (PCA) was performed as described in Opdam et al. 2017 [16]. For every MEM, a matrix showing if a reaction was present (1) or absent (0) was generated. Reactions that were present or absent in all observed models were removed, and row means of the matrix were zero-centered. Principal component analysis on reactions was then performed. Furthermore, the variance explained by each factor (MEM, diet, gender, genotype) within a principal component was calculated by taking the square of the maximum Pearson correlation coefficient (R^2^) of the component scores across all possible orderings of the factors as described in [16]. This was reported as a percentage. The validity of model separation observed in PCA was confirmed using t-distributed stochastic neighbour embedding (t-SNE), which is particularly suitable for high-dimensional data [49].

To assess the dynamical response of the models, we performed flux sampling using the artificial centering hit-and-run (ACHR) algorithm [50] on the extracted models. 1000 flux samples were generated for each of the models, and the mean flux of each reaction was used to identify the reactions that are either down- or up-regulated in pairwise comparisons of specific factors (diet, gender, and genotype) according to Spearman’s rank correlation. The reactions identified to be significantly changed were then used to perform the enrichment analysis of metabolic subsystems using the hypergeometric test. The obtained p-values were adjusted for multiple testing using the Benjamini-Hochberg procedure. The implementation of the described analysis is available as a set of IPython (IPYNB) notebooks at https://github.com/CompBioLj/GEMS_and_MEMS.

## 3 Results

### 3.1 Thresholds affect the extracted models

**Fig. 2:**
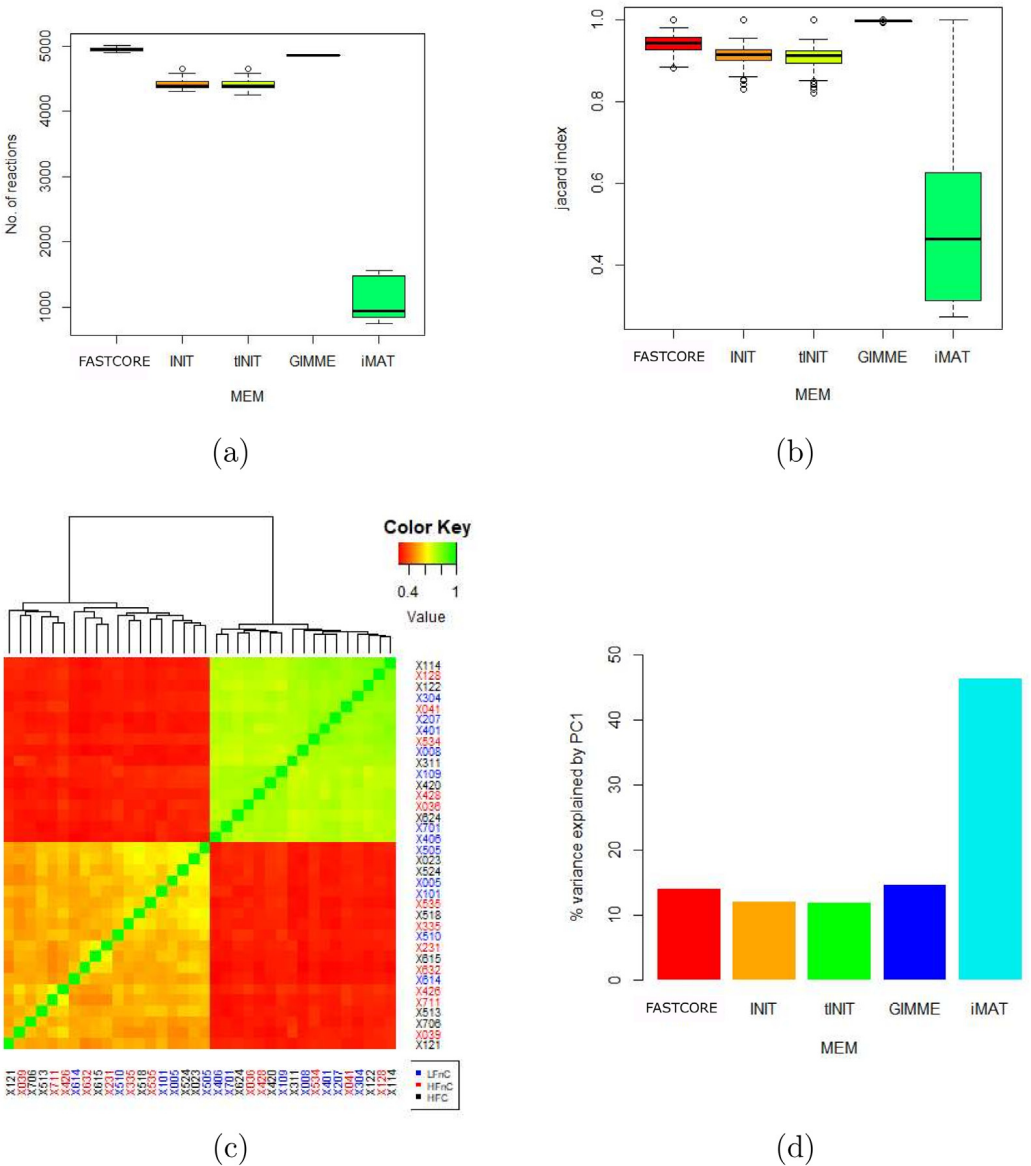
Comparison of models produced by different MEMs. Fig. 2 (a) summarizes the size of the models, (b) summarizes the Jaccard indices, (c) shows the Jaccard indices of models produced by iMAT, and (d) shows the variance explained by PC1 of each MEM. PC1: first principal component; MEM: model ex.traction method.

Our results indicate that the models extracted with the iMAT methodology were able to explain the highest amount of variance in comparison to other model extraction methods (see Section 3.2 and Fig. 2d). We thus opted to use the iMAT GEMs to assess the impact of thresholding on the extracted models. Two types of thresholds were used. First, thresholds were considered for each sample. This was done by taking a certain percentile of all the data within each sample to get the cutoff for highly expressed genes in that sample. This was achieved by taking a certain percentile of data to get the cutoff for highly expressed genes in that sample. The $50^{\mathrm{th}},70^{\mathrm{th}},75^{\mathrm{th}}$ and $80^{\mathrm{th}}$ percentile were considered. With the exception of models extracted at the $50^{\mathrm{th}}$ percentile threshold, the range and distribution of the Jaccard index and the size of the models were generally similar (see Figs. 1a and b). The percentage of variance explained by the PC1 was the smallest at the $50^{\mathrm{th}}$ percentile threshold but similar for other threshold values (see Fig. 1c).

The second type of thresholds was set per gene, i.e., by taking a percentile per gene for all samples. In this case the same threshold values were used for all samples, but were different among different genes. We considered the $80^{\mathrm{th}},90^{\mathrm{th}}$ and $95^{\mathrm{th}}$ percentile gene expression for each gene as a cutoff to classify if a gene is highly expressed or not. We also considered taking the mean of each gene as a cutoff for the same purpose. From the perspective of principal component analysis, the highest variance explained by PC1 was 17% (see Fig. 1d). Considering thresholds at $80^{\mathrm{th}},90^{\mathrm{th}}$ and $95^{\mathrm{th}}$ percentile, the highest variance explained by PC1 was achieved at the $90^{\mathrm{th}}$ percentile threshold.

PC1s from models where the threshold was taken per sample explained more variance than from models where the threshold was taken per gene. In general, the choice of thresholding strongly affected the content of the extracted models and their ability to capture variance in the data. Thresholding per sample with an $80^{\mathrm{th}}$ percentile as a cutoff value was considered as the most appropriate and was used in further analyses.

### 3.2 Extracted models vary with algorithm

Context-specific models were extracted using the GIMME, iMAT, FASTCORE, INIT, and tINIT model extraction methods (MEMs). We performed the extraction process using the COBRA [51], [52] and RAVEN Toolboxes [53] in Matlab R2019b (MathWorks Inc., Natick, Massachusetts, USA) using the Gurobi solver [54].

The number of remaining reactions in an extracted model was considered to represent the size of a model. Different MEMs generated models of different sizes (see Fig. 2a). iMAT-produced models were significantly different in size from other MEMs (t-test: p-value < 0.001). For each model within each MEM, we identified reactions that were present (1) or absent (0) and calculated the Jaccard index between all possible pairwise combinations to compare the similarities between models. For GIMME, FASTCORE, tINIT, and INIT, the Jaccard index was between 0.8 and 1.0, implying that these models are very similar (see Fig. 2b). For iMAT, the Jaccard index ranged from 0.27 to 1.0, indicating that GEMs extracted with iMAT varied substantially (see Figs. 2 b,c). Furthermore, we performed principal component analysis (PCA) on the matrix of reactions for each MEM. The PC1 explained the highest variance in iMAT derived models in comparison to PC1s of other MEMs (see Fig. 2d).

**Fig. 3:**
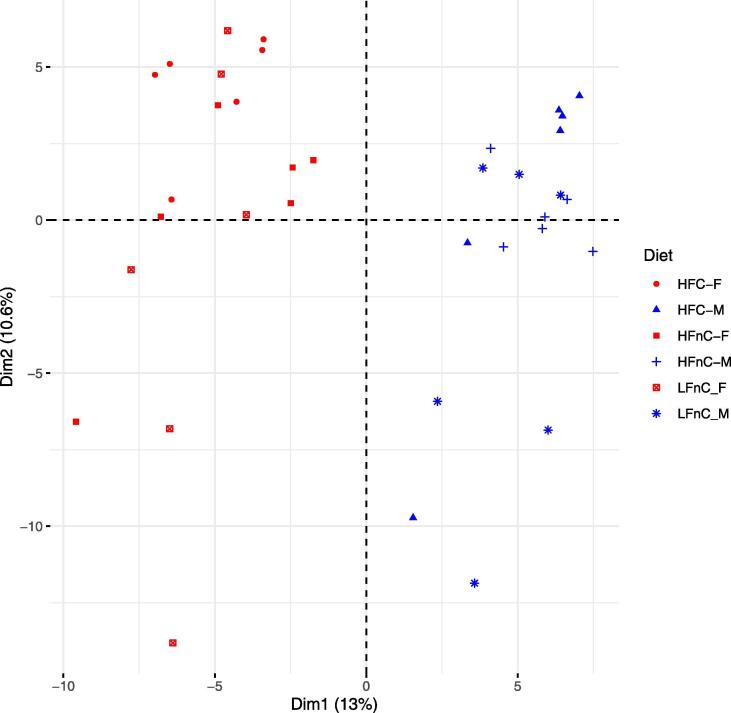
PCA plot showing separation by gender performed on the FASTCORE extracted models. Blue colour indicates male and red colour female samples, respectively. PCA: principal component analysis; F: female; M: male; LFnC: low fat without cholesterol; HFnC: high fat without cholesterol; HFC: high fat with cholesterol.

In addition, we analysed how well do the clusters observed in the feature space described by the first two principal components (PC1 and PC2) comply with the groups defined by the experiment, namely diet, gender, and genotype. The PCA plot (PC1 versus PC2) of models extracted with iMAT did not show consistent clustering of samples to predefined groups (see Supplementary Fig. 1). This was similar for GIMME (see Supplementary Fig. 2). Separation by gender was consistent with the clustering by PC2 of INIT explaining approximately 8% of variance, and at least partially consistent with the clustering by PC2 of tINIT explaining approximately 7.4% of variance in the data (see Supplementary Figs. 3 and 4). GEMs extracted using FASTCORE were appropriately clustered by gender by the PC1 explaining approximately 13% of variance in the data (see Fig. 3 and Supplementary Fig. 5). All PCA plots performed on the FASTCORE extracted models are available as Supplementary Fig. 5.

To verify the validity of the clustering obtained using PCA, we additionally performed t-distributed stochastic neighbour embedding (t-SNE) using different values of perplexity parameter [49]. Consistent clustering was obtained when the perplexity value was at least 15. The observed separation of models complied with the PCA results (see Supplementary Figs. 6 and 7). Separation of models was consistent with gender in FASTCORE, INIT and tINIT, whereas using the PCA separation by gender was observed in FASTCORE and INIT and partially also in tINIT. Clustering by gender and only by gender was also observed in the original study [46], which confirms the adequacy of our analysis.

**Fig. 4:**
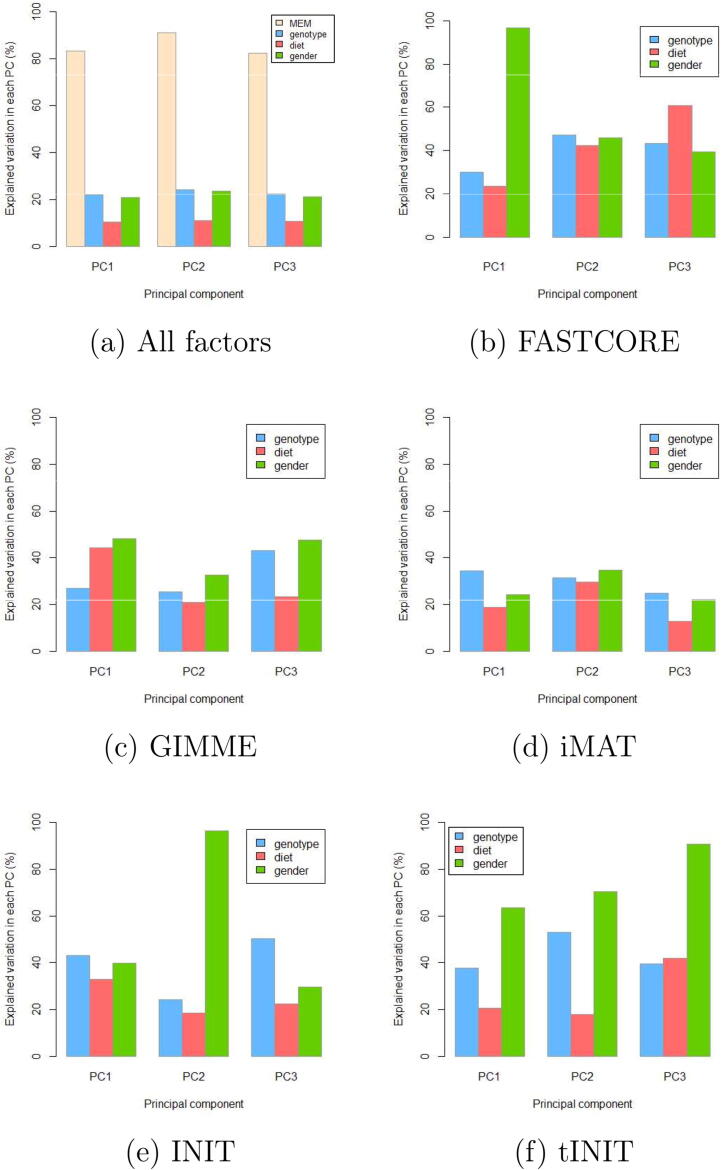
Contribution of different factors, namely, MEM (yellow), diet (red), genotype (blue) and gender (green) to PC1, PC2 and PC3. Fig. 4 (a) shows the contribution of all factors. Remaining figures show the contribution of diet, gender and genotype in models extracted with FASTCORE (b), GIMME (c), iMAT (d), INIT (e) and tINIT (f). MEM: model extraction method; PC: prin.cipal component.

We further assessed how much each of the factors (MEM, diet, gender and genotype) contributes to the variation explained by the first three principal components (PCs). This was evaluated by taking each factor and calculating the squares of Pearson correlation coefficient (R^2^) of component scores across all possible orderings of the factors [16]. Considering all factors, the selection of a MEM explains the most variability in the PCs (Fig. 4a). Furthermore, in FASTCORE (Fig. 4b) and and GIMME (Fig. 4c) extracted models, gender explained the most variability in the PCs as was also observed in the original study [46].

In the models extracted with iMAT (Fig. 4d), the observed three factors, namely, gender, genotype, and diet, generally contributed equally with genotype having a slight edge in comparison to diet and gender. Gender explained the most variability in models extracted with INIT and tINIT (Fig. 4e,f).

### 3.3 Enrichment analysis of metabolic subsystems

The models extracted with FASTCORE were able to capture the largest amount of true variability of the observed data (see Fig. 3). We thus opted to perform further analysis only on these models. Since the basic version of flux balance analysis (FBA) is unable to provide unique solutions, we opted to analyse the activity of the observed metabolic reactions using flux sampling using the artificial centering hit-and-run (ACHR) algorithm [50]. It was performed on the models extracted with the FASTCORE algorithm to obtain the mean values of reaction fluxes in each of the models. We performed the flux sampling using COBRA [51], [52] and the Gurobi solver [54] in Matlab R2019b.

**Fig. 5:**
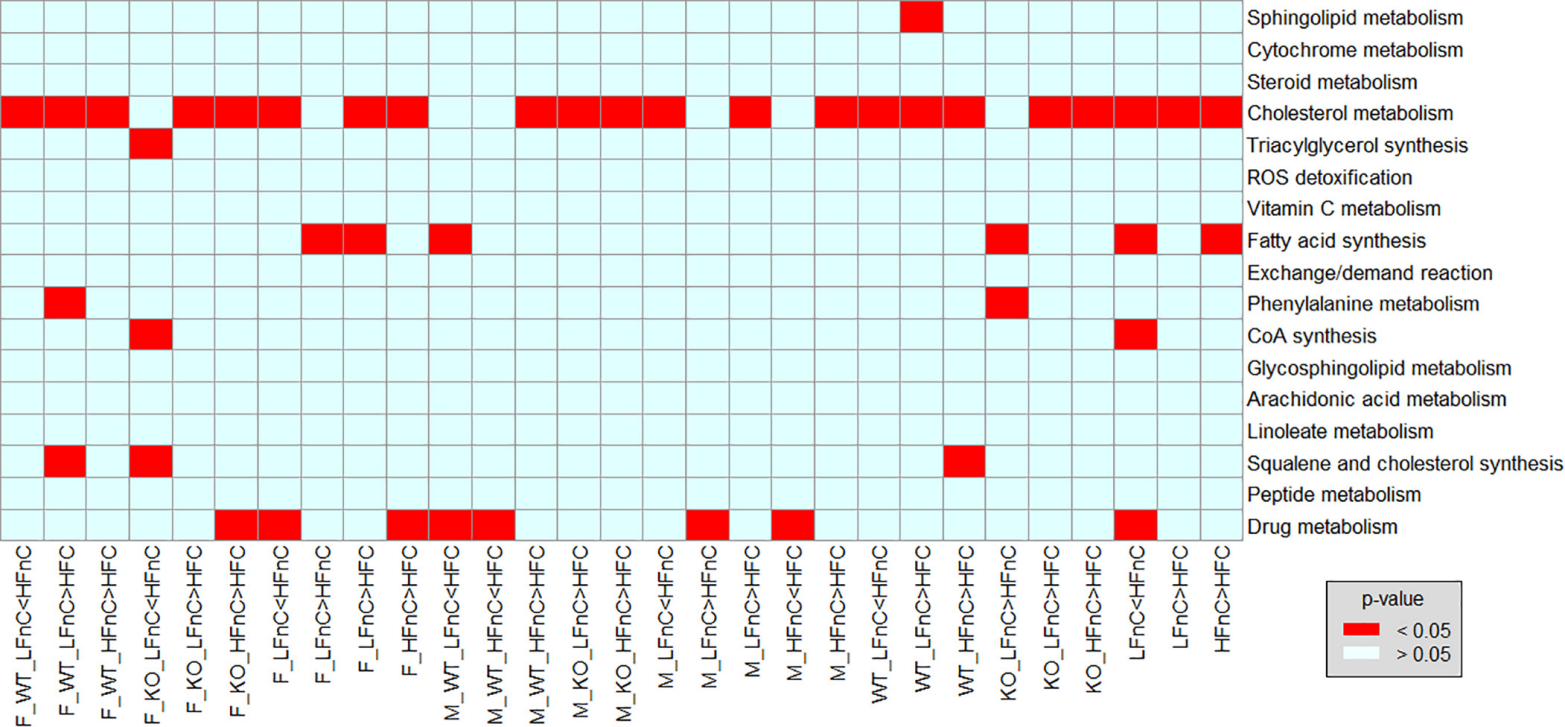
Metabolic subsystems enrichment analysis between different diets. Subsystems that were differentially enriched between two groups and those associated with cholesterol are shown. Cholesterol metabolism was enriched in mice on HFnC diet compared to HFC diet. The less than symbol (<) between the groups corresponds to down-regulation and the more than symbol (>) corresponds to up-regulation. F: female; M: male; WT: wild type; KO: knockout; LFnC: low fat without cholesterol; HFnC: high fat without cholesterol; HFC: high fat with cholesterol.

We identified up- and down-regulated reactions between different groups of extracted models (e.g., wild type versus knockout groups) and their combinations using Spearman’s rank correlation. Genome-scale metabolic models are usually composed of several subsystems containing metabolic reactions with a specific function (e.g. cholesterol synthesis subsystem) [52], [53]. Based on the identification of differentially regulated reactions, we further analysed the enriched subsystems between different groups using the hypergeometric test. We compared the models on the basis of diet (see Fig. 5).

To check if the obtained models comply with our expectations, we further observed how different diets affect the cholesterol synthesis and metabolism subsystems. Fig. 5 shows that cholesterol metabolism was enriched in mice on LFnC or HFnC diet compared to HFC diet. However, cholesterol metabolism was down-regulated in mice on LFnC diet compared to HFnC diet. Furthermore, Cholesterol synthesis was up-regulated in female wild type mice on LFnC diet compared to the HFC diet. In female knockout mice, cholesterol synthesis was down-regulated in mice on the LFnC diet in comparison to the mice on the HFnC diet. In wild type mice, cholesterol synthesis was up-regulated in mice on the HFnC diet compared to the HFC diet. There was no significant subsystem enrichment due to genotype or gender alone. A 5% level of significance after the adjustment for multiple testing (p-value: < 0.05) was considered in all analyses.

## 4 Discussion

**Fig. 6:**
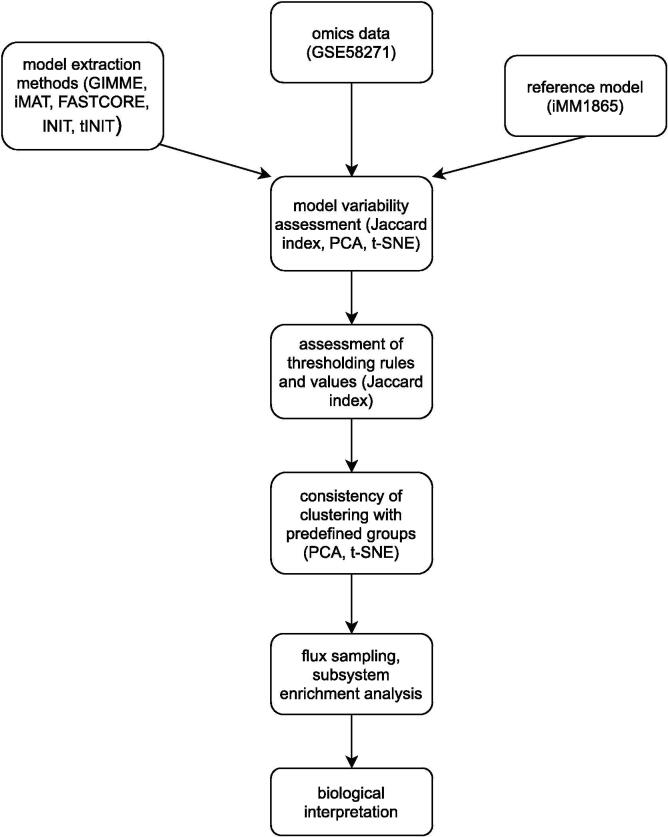
Overview of the proposed methodology. PCA: principal component analysis; t-SNE: t-distributed stochastic neighbour embedding.

The aim of this work was to highlight the factors that have the strongest influence on the context-specific extraction of genome-scale metabolic models, especially in relation to the model-based analysis of omics data. Additionally, we proposed a methodology that could be followed in such analyses (see Fig. 6). This is composed of the following steps: (1) identification of the most suitable reference model; (2) extraction of context-specific GEMs using different MEMs with different configurations; (3) identification of the MEM and its configuration that is able to capture the variance of the observed data as well as the groups defined in the experiment; (4) analysis of obtained models using different approaches, such as PCA, t-SNE, and metabolic subsystems enrichment analysis.

We extracted GEMs using five model extraction methods i.e. GIMME, iMAT, FASTCORE, INIT and tINIT. The results show that the choice of a MEM affects the structure and contents of the output models. Additionally, the models varied greatly in size between MEMs. INIT and tINIT are very similar algorithms and so are their output models. These results are consistent with published reports [16], [17], [18]. We used Jaccard index to assess how much the models differed within each MEM and PCA to assess how much variance in the data each MEM was able to capture. GIMME produced very similar models and captured the least variability in the data. iMAT captured the most variability in the data (46% by PC1) but was unable to capture the true variability in the experiment. However, FASTCORE with 13% of variance explained by PC1 in this example was able to capture the largest amount of variability due to any of the observed factors, namely gender. Similar clustering was also observed in the original study [46]. This implies that it is important to select a MEM that not only captures the largest variance in the data, but also captures the groups that are aligned with predefined experimental groups. These groups could reflect different metabolic signatures and thus have a potential to guide downstream analyses. Our findings assert that the choice of a MEM greatly affects the output models.

We further assessed the impact of using different thresholds on the output models. Thresholds were set on gene expression data to identify highly expressed genes. For each model extracted with iMAT, we applied two kinds of thresholding, i.e., within a sample and within a gene. Thresholding within a sample captured unique individual differences in expression even if individuals were from the same experimental group. We see that the type of thresholding applied and the threshold values used strongly affect the output models.

We performed subsystem enrichment analysis to assess how cholesterol metabolism and synthesis varied between the groups separated by different diets. This is because knocking out the *Cyp51* gene interrupts cholesterol synthesis and metabolism [46], [55], [56], [57]. In our analysis, models extracted with FASTCORE showed that cholesterol metabolism significantly changed between groups with different diets. Cholesterol metabolism was enriched in mice on LFnC or HFnC diet compared to HFC diet. However, cholesterol metabolism was down-regulated in mice on LFnC diet compared to HFnC diet.

The proposed methodology, like other analyses using GEMs, requires selection of a suitable reference model. The choice of a reference model strongly affects the output models. The model should optimally describe the same tissue or at least the same organism as the samples used in the experiment. Moreover, the model should contain accurate gene-product-reaction (GPR) rules, which are used by MEMs to connect the omics data with metabolic reactions. In our case, we used the most recently published mouse model, namely iMM1865 [47].

Different MEMs implement different algorithms for data integration. As such, the output models differ greatly in their contents. In this analysis, we opted to use a selection of the state-of-the-art MEMs, namely, GIMME, iMAT, FASTCORE, INIT, and tINIT. However, other algorithms, such as CORDA [58], mCADRE [59] and MBA [60] could as well be applied. Selected MEMs also need to be configured as required by a particular algorithm, e.g., with the identification of the most suitable thresholding rules and values. The choice of values for a particular configuration needs to be guided by known knowledge and further analyses performed on selected MEMs. Identification of the most suitable MEM(s) needs to be based on the results from the analysis of the obtained models using different approaches, such as PCA, t-SNE, various distance metrics, and metabolic subsystem enrichment analysis. Ideally, a MEM of choice will explain the most variance in the observed dataset and will be able to separate the models in compliance with the groups defined in the experiment. Moreover, the obtained results of further analyses of the models extracted with the selected MEM will reflect the biological relevance and will provide a platform to generate novel knowledge and hypotheses. Such an ideal situation may not be obtained easily. When compromises are required, the MEM that best captures the groups defined by the experiment should be selected. In this context, the MEM that has the largest percentage of explained variance within the PC1 for a selected group should be selected (see Fig. 4).

Even though the proposed methodology was demonstrated using only transcriptomics data, different omics data could as well be used in the process. For example, metabolomics and lipidomics data can be mapped into the context of metabolic reactions [61]. These can be used to identify a set of tasks or core reactions that need to be executed by a model. Moreover, specific model extraction methods, such as tINIT, allow a direct integration of non-transcriptomics data. Namely, tINIT algorithm accepts a list of metabolites the model should be able to produce [45].

We propose that the analysis of omics data using GEMs should be initiated with an extraction of different context-specific models using different MEMs. This should be followed with the application of various thresholding rules and different threshold values in the process of reconstruction. The resulting models should then be analysed to select the most appropriate MEM(s), threshold values and thresholding rules that best capture the variability in the data while capturing the known experimental groups.

## CRediT authorship contribution statement

**Andrew Walakira:** Methodology, Software, Formal analysis, Investigation, Writing - original draft, Writing - review & editing, Visualization. **Damjana Rozman:** Conceptualization, Writing - review & editing, Funding acquisition. **Tadeja Režen:** Conceptualization, Writing - review & editing. **Miha Mraz:** Writing - review & editing, Funding acquisition, Supervision. **Miha Moškon:** Conceptualization, Methodology, Software, Formal analysis, Investigation, Writing - review & editing, Supervision.

## Declaration of Competing Interest

The authors declare that they have no known competing financial interests or personal relationships that could have appeared to influence the work reported in this paper.
