# Supplementary material for: Guided extraction of genome-scale metabolic models for the integration and analysis of omics data
Source: Comput Struct Biotechnol J. 2021 Jun 8;19:3521–30. doi: 10.1016/j.csbj.2021.06.009 (PMC8225705; doi:10.1016/j.csbj.2021.06.009)
Supplement: Supplementary file 1 [file mmc1.pdf]

Supplementary text for:

Guided extraction of genome-scale metabolic models for the integration and analysis of omics data

Andrew Walakira[1], Damjana Rozman[1], Tadeja Režen[1], Miha Mraz[2], and Miha Moškon[2*]

[1]Centre for Functional Genomics and Bio-Chips, Institute for Biochemistry and Molecular Genetics, Faculty of Medicine, University of Ljubljana, Ljubljana, Slovenia

[2]Faculty of Computer and Information Science, University of Ljubljana, Slovenia

[*] corresponding author
E-mail: miha.moskon@fri.uni-lj.si

1 Supplementary Figures

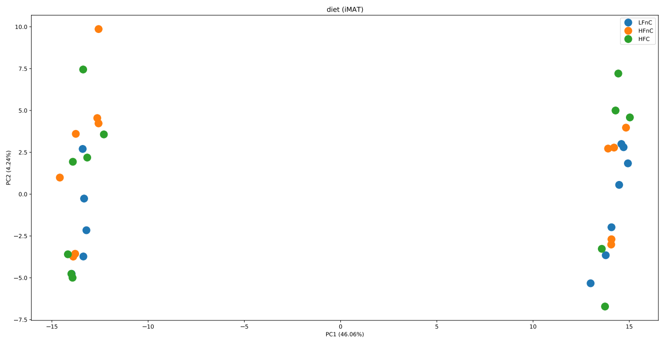

(a)

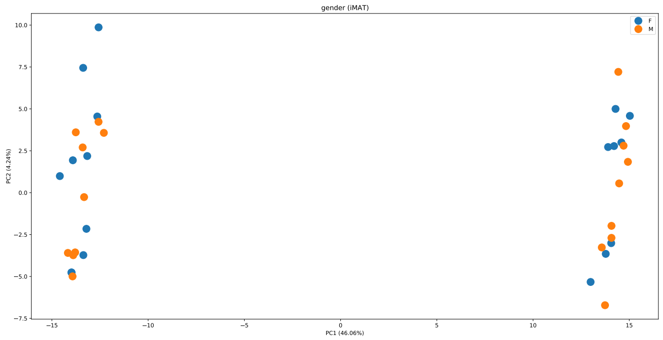

(b)

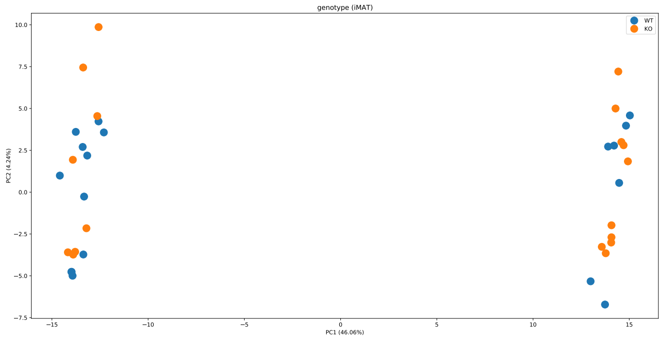

(c)

Supplementary Figure 1: PCA plot showing separation by diet (a), gender (b) and genotype (C) performed on the iMAT extracted models. There is no separation by any of the experimental groups.

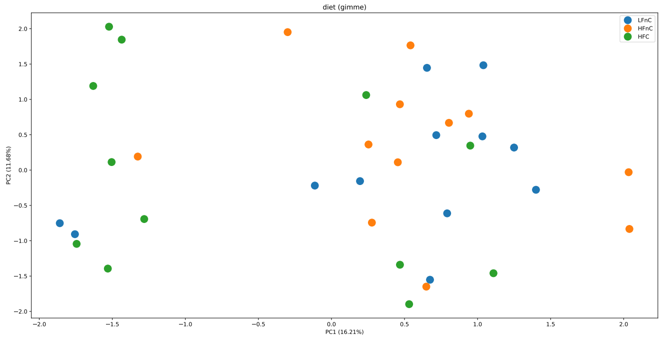

(a)

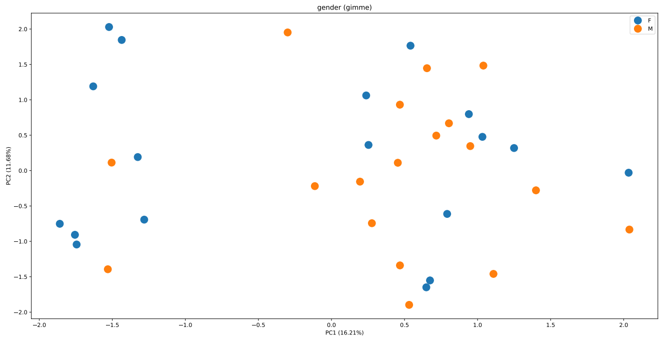

(b)

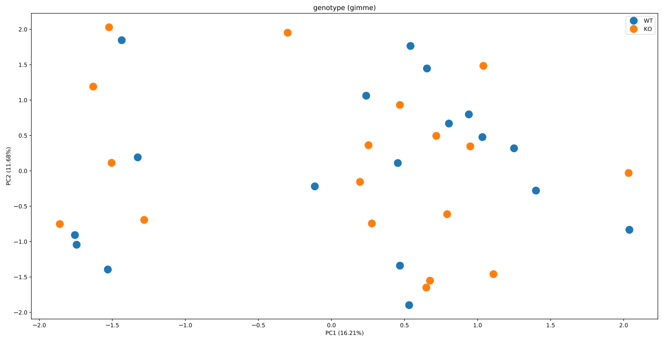

(c)

Supplementary Figure 2: PCA plot showing separation by diet (a), gender (b) and genotype (C) performed on the GIMME extracted models. There is no separation by any of the experimental groups.

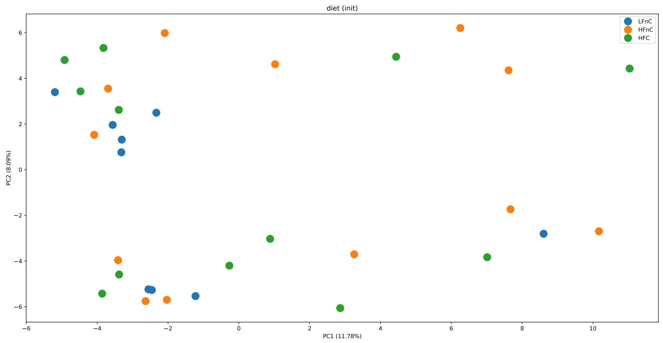

(a)

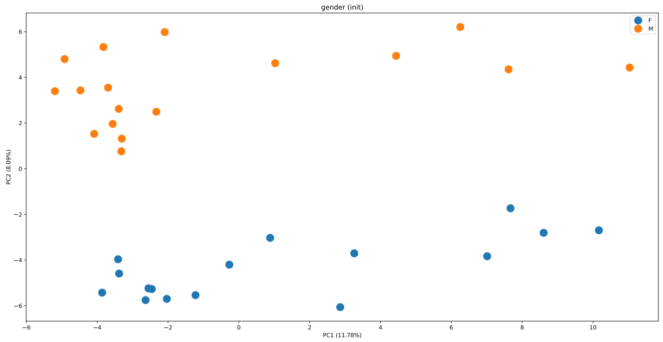

(b)

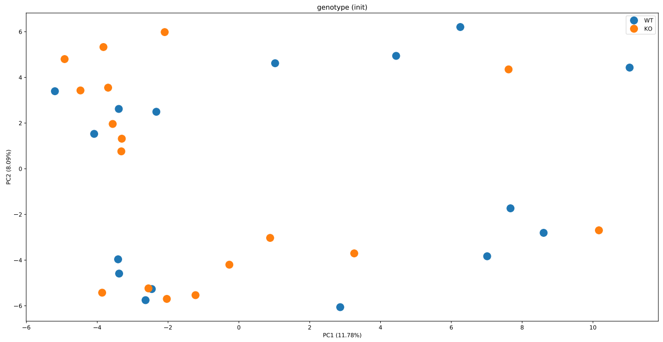

(c)

Supplementary Figure 3: PCA plot showing separation by diet (a), gender (b) and genotype (C) performed on the INIT extracted models. INIT separated samples by gender on PC2 but the variance explained by PC2 was only 8%. There is no separation by any other of the experimental groups

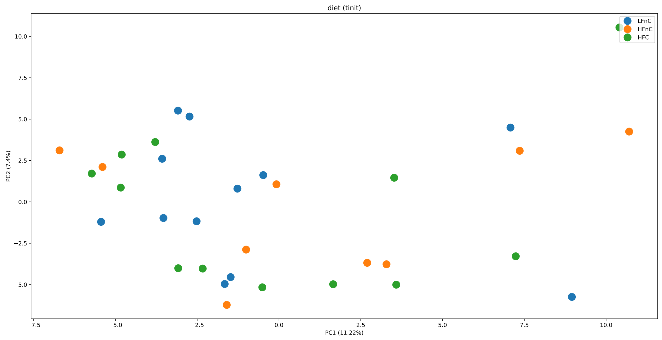

(a)

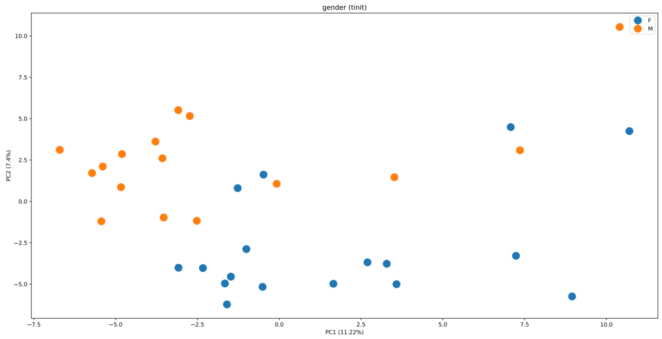

(b)

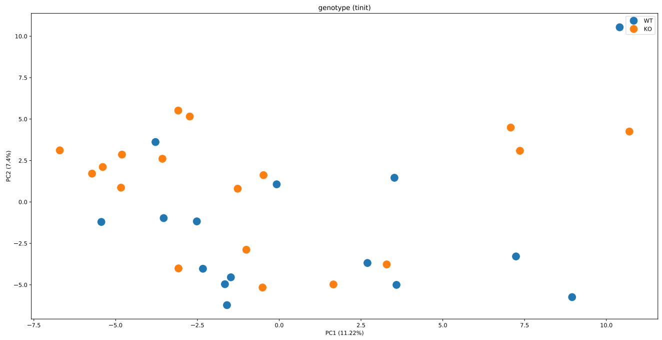

(c)

Supplementary Figure 4: PCA plot showing separation by diet (a), gender (b) and genotype (C) performed on the tINIT extracted models. There is no separation by any of the experimental groups.

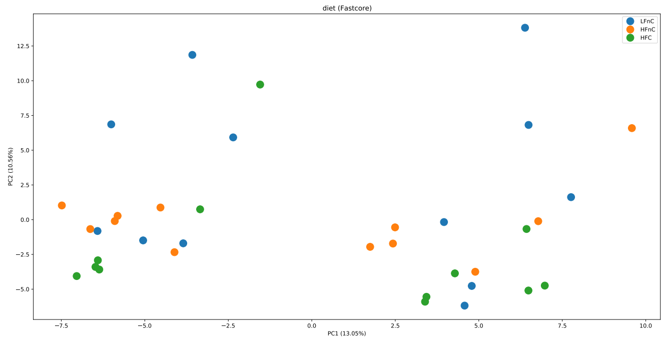

(a)

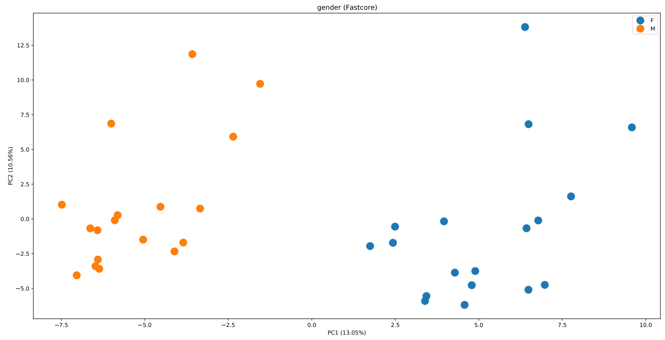

(b)

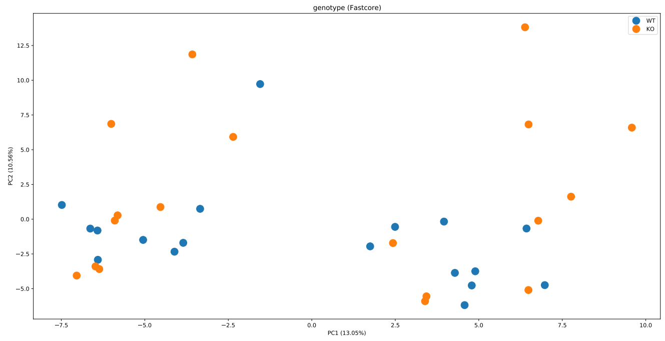

(c)

Supplementary Figure 5: PCA plot showing separation by diet (a), gender (b) and genotype (c) performed on the FASTCORE extracted models. The models separated well based on gender (b), but there was no separation by diet (a) and genotype (c).

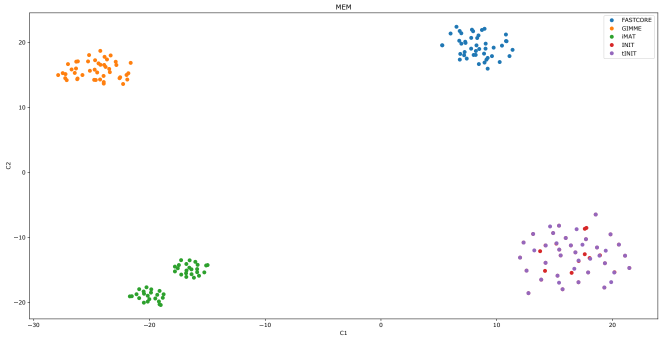

Supplementary Figure 6: t-SNE plot showing separation by model extraction method (MEM). The figure was generated with the perplexity value set to 30. The models are mostly well separated on MEM except for INIT and tINIT produced models.

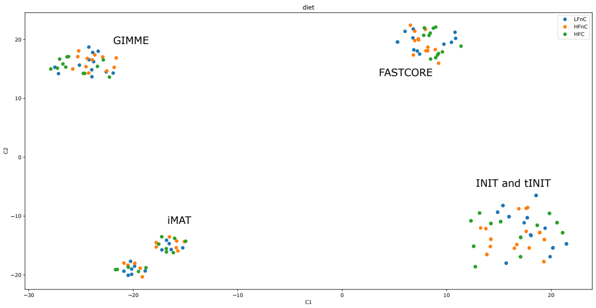

(a)

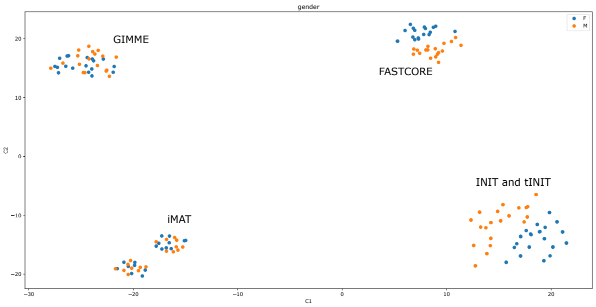

(b)

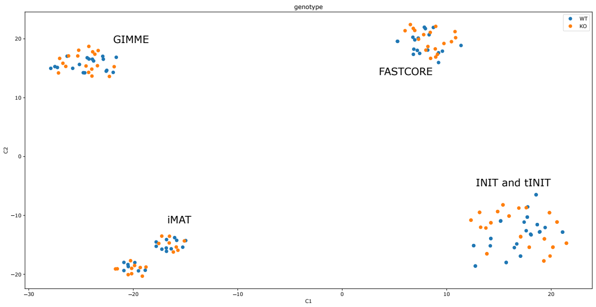

(c)

Supplementary Figure 7: t-SNE plot showing separation by diet (a), gender (b) and genotype (c) performed on all of the extracted models. The figures were generated with the perplexity value set to 30. FASTCORE, INIT and tINIT produced models are separated by gender. No separation was observed by diet or genotype in either of the MEMs.
